# Renal cell carcinoma: Impact of mode of detection on its pathological characteristics

**DOI:** 10.4103/0970-1591.57919

**Published:** 2009

**Authors:** Paresh Jain, R Surdas, Pallavi Aga, Manoj Jain, Rakesh Kapoor, Aneesh Srivastava, Anil Mandhani

**Affiliations:** Department of Urology and Kidney Transplantation, SGPGIMS, Lucknow, India

**Keywords:** Incidental, pathology, symptomatic

## Abstract

**Objective:**

Data correlating mode of presentation of renal cell carcinoma (RCC) with pathological prognostic factors is sparse from India. We compared RCC presenting incidentally with those presenting symptomatically with respect to pathological prognostic factors and assessed whether this could serve as a decision making resource for diagnosing small and more favorable tumors.

**Materials and Methods:**

The data were reviewed for 328 patients operated for renal tumors between January 2000 and October 2008 at our institute. The pathological factors (tumor size, stage, grade, histopathological type) in relation to the mode of presentation were analyzed according to 1997 TNM criteria. Statistical analysis was performed via the chi-square (Fisher exact) and Mann - Whitney *U* test. The statistical significance level utilized was *P* < 0.05.

**Results:**

Among the patients assessed, 93 (28.4%) had incidental diagnosis and 235 (71.6%) had symptomatic presentation. Sex and side distribution was not significantly different in the two groups. Mean tumor size was 5.75 ± 2.73 cm in incidentally detected RCC (IRCC) and 9.32 ± 3.70 (*P* < 0.001) in symptomatic RCC (SRCC). Stage I and II tumors were significantly greater in IRCC than SRCC (*p*<0.001 and 0.005 respectively) whereas stage III and IV tumors were significantly less in IRCC than SRCC. There was a predominance of higher grade tumors in SRCC, 50% being higher grades (Fuhrman's grade III and IV) in SRCC than 28.1% in IRCC (*P* = 0.003). There were 4 tumors with collecting duct histology in SRCC and none in IRCC. Sarcomatoid differentiation was present in 14 and 1 patient in SRCC and IRCC respectively.

**Conclusion:**

Incidental detection of renal carcinoma as compared to symptomatic tumors is lower in India as compared to western world. Incidental tumors have significantly favorable pathological prognostic factors. Our results might form a basis for further studies on how to pick RCC at an earlier stage.

## INTRODUCTION

In recent years, incidental detection of renal cell carcinoma (RCC) has increased with the mainstream use of abdominal computerized tomography (CT) and ultrasound.[1–7] Studies from western countries have demonstrated that incidentally discovered renal cell carcinoma (IRCC) tends to be smaller in size, of lower stage, and results in better survival outcomes than that of symptomatic RCC (SRCC).[8–10] In corollary to this, many have proposed screening for RCC.[11–14] There is paucity of data for Indian patients regarding IRCC and its pathological characteristics. Herein, we present our data on mode of detection and pathological features of IRCC as compared to SRCC.

## MATERIALS AND METHODS

Records of 328 consecutive patients operated for clinically suspected renal cell carcinoma between January 2000 and November 2008 were reviewed from the computer based hospital information system, patient charts and pathology data-base.

All the patients had contrast-enhanced CT and or MRI apart from the metastatic workup whenever needed. Kidney tumors with a preoperative diagnosis of angiomyolipoma and transitional cell carcinoma were excluded. Renal cell carcinoma was considered to be incidental, when the diagnostic evaluation was not initiated secondary to any symptoms or signs associated with renal cell carcinoma. Lesions were considered to be symptomatic, SRCC, when patients presented with symptoms due to the tumor itself (e.g. flank mass, flank pain and hematuria) or due to advanced disease (such as weight loss or fever).

Patient's age, sex, symptoms at presentation, tumor size, tumor stage, (TNM 2002) histopathological type and grades were compared between IRCC and SRCC.

SPSS 16.0 software was used for statistical analysis. All the categorical variables were summarized using frequencies and percentages. The chi square (Fisher exact) test and Mann-Whitney *U* - test were used to compare the clinicopathologic characteristics between IRCC and SRCC. p-value of less than 0.05 was considered significant.

## RESULTS

Among the total of 328 patients operated for renal cell carcinoma, 93 (28.35%) were detected incidentally and 235 (71.64%) with symptoms pertaining to the renal cell carcinoma. Of the 235 symptomatic patients 48.1% presented with hematuria, 31.9% with flank pain and 14.5 % with a flank mass. Table 1, lists patient and tumor characteristics in the symptomatic and incidental groups.

**Table 1:** Demographic and pathological characteristics of incidental and symptomatic renal cell carcinoma

	Incidental	Symptomatic	*P* value
No. of patients	93	235	
Mean age (year) SD	52.31	52.30	0.927
Male (%)	73.1	80.7	1.00
Female (%)	26.9	18.3	
Right (%)	57	58.7	0.507
Center (%)	41.9	41.3	
Mean tumor size (cm)	5.753	9.327	<.001
T Stage (%)			
I	65.6	21.1	<0.001
Ia	31.2	1.3	<0.001
Ib	34.4	19.8	0.006
II	20.4	36.3	0.005
III	12.9	30.9	0.001
IV	1.1	11.7	0.001
Grade (%)			
Low (grade 1 and 2)	71.9	50	0.003
High (grade 3 and 4)	28.1	50	0.003

Patient's age and sex distribution were similar in both the groups. There was significant difference in the tumor size between the symptomatic (mean 5.75 ± 2.73) and incidentally detected RCC mean (9.32 ± 3.70) (p < 0.001). [Table 1] On comparison of the T stage, it was found that proportion of higher stage tumors were more in symptomatic RCC [Table 1]. Similarly there was predominance of higher grade tumors in SRCC, 50% being higher grades (Fuhrman's grade III and IV) in SRCC than 28.1% in IRCC (p=.003). [Table 1]

Although there was no difference in the incidence of common histopathological types of the RCC in both the groups, poor risk characteristics were more common in SRCC. [Table 2] None of the patients in incidentally detected RCC had collecting duct tumor and sarcomatoid differentiation was present in only 1 patient whereas; in the symptomatic group there were 4 collecting duct tumors and 14 patients had sarcomatoid differentiation.

**Table 2:** Histopathological types in IRCC and SRCC

Histopathological types	Incidental	Symptomatic
Clear cell	59 (63.4)	162 (68.9)
Papillary	15 (16.1)	26 (11.0)
Chromophobe	6 (6.5)	8 (3.4)
Oncocytoma	6 (6.5)	4 (1.7)
Collecting duct	0	4 (1.7)
Sarcomatoid	1 (1.07)	14 (6.0)
Miscellaneous	6 (6.5)	17 (7.2)

After analyzing the reasons for consultation sought by patients with incidental diagnosis, it was found that a significant proportion (26.8%) of patients had lower urinary tract symptoms (LUTS) for which an ultrasound was done. [Table 3] Similar number of patients had nonspecific gastrointestinal symptoms as their presenting complaints. Other reasons for which ultrasonography picked up the tumor are given in Table 3.

**Table 3:** Indication of usg in patients detected incidentally

Reasons for detection	Number of patients (%)
Non specific gi symptoms	25 (26.88)
Lower urinary tract symptoms	25(26.88)
Menstrual complaints	5(5.3)
Cardiac evaluation	6(6.4)
Routine health check up	5(5.3)
Evaluation for diabetes	5(5.3)
Raised creatinine	5(5.3)
Headache / vertigo	3(3.2)
Post operative follow up (submandibular mass, retroperitoneal benign mass)	3(3.2)
Pedal edema	3(3.2)
Donor evaluation	2(2.1)
Scrotal pain	2(2.1)
Pregnancy	1(1.07)
Pleural effusion	1(1.07)
Contra-lateral pathology (UPJ obstruction and renal stone)	2(2.1)

## DISCUSSION

There has been an upsurge in the incidental detection of renal masses in western countries due to a widespread use of ultrasonography and cross sectional imaging.[1–7] Rates of incidental detection as per the current literature are up to 60% or even greater.[5]

There are no data from India on IRCC. With increasing awareness about health and burgeoning centers for ultrasonography there could be a possibility of an increasing trend in detecting IRCC. One study from our institute previously reported the rate of IRCC as 8%, which has increased to 28% in present study.[15] This might reflect the changing trends in our country too but the pace and the magnitude of change is not as much as seen in the west. Similarly the mean size of IRCC was much greater than what it has been reported from the western countries. This could be explained by lack of mandatory health insurance policy for masses and access to medical care for minor illnesses. Even executive health checkups are in scarcity.

Size, stage and grade are the most important prognostic factors for RCC. Most of the recent series have demonstrated incidentally detected tumors to be of smaller size, lower stage and lesser grade as compared to symptomatic tumors and thereby imparting an indirect survival advantage.[4,6,10,16–18]

When there is so much written about the IRCC then we need to answer one basic question and that is; whether IRCC is biologically different? Several studies have supported this argument that IRCC is an independent prognostic factor for survival signifying their less aggressive biological behavior.[4,10,19] Contrary to this, studies have also demonstrated that incidental diagnosis is not an independent prognostic factor on multivariate analysis, the improved survival is because of tumors being diagnosed at a lower stage and grade than symptomatic tumors.[18,20]

We noted that incidental tumors were of significantly lower stage, lower grade and better histopathological characteristics. In our experience more aggressive histology, such as sarcomatoid variants, collecting duct tumors and high grade tumors were more often seen in symptomatic tumors.

Although assessing survival was not a part of our study, but looking to the significantly lower stage and grade in IRCC, a better survival for this group of patients could be contemplated.

The increased detection of incidental renal cell carcinoma by ultrasound as well as the relatively favorable prognostic factors of such lesions, as reported in this and previous studies, has led some to consider using ultrasonography as a screening tool.[11] However, the low prevalence of the disease prevents population-based screening from being cost-effective.

Modified form of screening has been recommended in high risk groups such as those with acquired renal cystic disease, long-term dialysis, Von Hippel-Landau disease or age older than 50 years.[9,12] A case finding approach in which a brief examination of the kidneys should be done simultaneously with ultrasound performed for non-urological reasons was proposed by Thompson *et al*.[8] Similarly in a meta-analysis of 25 studies from Germany, it was recommended that though primary screening would be expensive, patients having ultrasonography for other reasons should have the retroperitoneum examined, especially if they are more than 45 years of age.[13]

Massod *et al*. in their study on incidental detection of RCC provided a good reason to offer renal tract ultrasonography for patients referred with LUTS.[14] They concluded that not only does it exclude hydronephrosis, but also provides an opportunity to detect coincidental cases of RCC.[14]

We also detected coexistent RCC in 26.8% patients evaluated for LUTS. Thus the American urological association guideline regarding the selective use of ultrasonography in patients undergoing evaluation for LUTS in benign prostate hyperplasia with normal creatinine needs further justification in our set up. In countries like India where routine health check up examinations are seldom done, offering an opportunistic screening by ultrasonography to the patients presenting to the clinician, would be a moot point to be clarified further.

The drawback of our study is that survival analysis was not done though it definitely would have strengthened our study, this was because we did not have the complete follow up of our patient population.

## CONCLUSION

Incidentally detected renal cell carcinoma has significantly better pathological prognostic factors than SRCC. Symptomatic tumors present at a significantly higher stage and grade, and are substantially more aggressive than incidental lesions, particularly at later stages. Until more effective treatment modalities become available for advanced stage RCC, it would be better to detect them at an early stage. Although a population based screening would not be an answer to detect RCC at an early stage, the role of opportunistic screening of patients presenting to the clinician by doing an ultrasonography is to be explored.

## References

[1] Konnak JW, Grossman HB (1985). Renal cell carcinoma as an incidental finding. J Urol.

[2] Ueda T, Mihara Y (1987). Incidental detection of renal cell carcinoma during radiological imaging. Br J Urol.

[3] Smith SJ, Bosniak MA, Megibow AJ, Hulnick DH, Horii SC, Raghavendra BN (1989). Renal cell carcinoma: earlier discovery and increased detection. Radiology.

[4] Bretheau D, Lechevallier E, Eghazarian C, Grisoni V, Coulange C (1995). Prognostic significance of incidental renal cell carcinoma. Eur Urol.

[5] Jayson M, Sanders H (1998). Increased incidence of serendipitously discovered renal cell carcinoma. Urology.

[6] Luciani LG, Cestari R, Tallarigo C (2000). Incidental renal cell carcinoma-age and stage characterization and clinical implications: study of 1092 patients (1982-1997). Urology.

[7] Leslie JA, Prihoda T, Thompson IM (2003). Serendipitous renal cell carcinoma in the post-CT era: continued evidence in improved outcomes. Urol Oncol.

[8] Thompson IM, Peek M (1998). Improvement in survival of patients with renal cell carcinoma: the role of the serendipitously detected tumor. J Urol.

[9] Ebert T, Owusu G, Strotmann P (1999). Do we need screening for renal cell carcinoma (RCC)?. J Urol.

[10] Patard JJ, Rodriguez A, Rioux-Leclercq N, Guillé F, Lobel B (2002). Prognostic significance of the mode of detection in renal tumours. BJU Int.

[11] Tosaka A, Ohya K, Yamada K, Ohashi H, Kitahara S, Sekine H (1990). Incidence and properties of renal masses and asymptomatic renal cell carcinoma detected by abdominal ultrasonagraphy. J Urol.

[12] Malaeb BS, Martin DJ, Littooy FN, Lotan Y, Waters WB, Flanigan RC (2005). The utility of screening renal ultrasonography: identifying renal cell carcinoma in an elderly asymptomatic population. BJU Int.

[13] Reu BJ, Rettenmaier G (1994). Der Nier en tumor als sonographiscter zufallsbefund. Ultraschal Med.

[14] Masood J, Lane T, Koye B, Vandal MT, Barua JM, Hill JT (2001). Renal cell carcinoma: incidental detection during routine ultrasonography in men presenting with lower urinary tract symptoms. BJU Int.

[15] Srivastava A, Mandhani A, Kapoor R, Jain M, Dubey D, Srivastava A (2004). Prognostic factors in patients with renal cell carcinoma: Is TNM (1997) staging relevant in Indian subpopulation?. Indian J Cancer.

[16] Tsui KH, Shvarts O, Smith RB, Figlin R, de Kernion JB, Belldegrun A (2000). Renal cell carcinoma: prognostic significance of incidentally detected tumors. J Urol.

[17] Dall'Oglio MF, Srougi M, Gonçalves PD, Leite K, Nesrallah L, Hering F (2002). Incidental and symptomatic renal tumors: impact on patient survival. Sao Paulo Med J.

[18] Gudbjartsson T, Thoroddsen A, Petursdottir V, Hardarson S, Magnusson J, Einarsson GV (2005). Effect of incidental detection for survival of patients with renal cell carcinoma: results of population-based study of 701 patients. Urology.

[19] Lee CT, Katz J, Fearn PA, Russo P (2002). Mode of presentation of renal cell carcinoma provides prognostic information. Urol Oncol.

[20] Bulnes Vázquez V, Alvarez-Múgica M, Fernández Gómez JM, Nava Tomás E, Jalón Monzón A, Meilán Martínez A (2008). Clinicopathological features of renal cell carcinoma incidentally detected through radiological studies. Actas Urol Esp.

